# Rising rates of recent preexposure prophylaxis exposure among men having sex with men newly diagnosed with HIV: antiviral resistance patterns and treatment outcomes

**DOI:** 10.1097/QAD.0000000000003143

**Published:** 2021-12-06

**Authors:** Nicolò Girometti, Sheena McCormack, Victoria Tittle, Alan McOwan, Gary Whitlock

**Affiliations:** a 56 Dean Street, Chelsea & Westminster Hospital NHS Foundation Trust; b MRC Clinical Trials Unit at UCL, London, UK.

**Keywords:** antiviral resistance, HIV, men having sex with men, preexposure prophylaxis

## Abstract

**Introduction:**

Preexposure prophylaxis (PrEP) is contributing to achieve a reduction in HIV diagnoses in men having sex with men (MSM). Albeit infrequent, HIV infections in the context of recent PrEP exposure represent a clinical challenge.

**Methods:**

Data on recent PrEP use and possible reasons leading to HIV infection were analysed in individuals newly diagnosed with HIV at 56 Dean Street clinic in 2016–2020. Demographics, immune-virological parameters, genotypic resistance test results and treatment management in this group were compared with those not reporting recent PrEP exposure using Mann–Whitney *U* test and Fisher's exact test.

**Results:**

Fifty-two of 1030 (5%) individuals reported recent PrEP exposure at HIV diagnosis; 98% were MSM, median age 34 years (interquartile range [IQR] 28–42), 65% of white ethnicity, 65% non-UK-born. 35% reported PrEP intake the day before testing HIV positive, 46% reported sub-optimal PrEP adherence since their last negative HIV test result. Thirty-three of 52 (63%) were self-sourcing PrEP and 9/52 (17%) reported issues with its supply. Recent PrEP use was associated to lower HIV viral load and higher CD4^+^ cell count at baseline than in counterparts non-recently exposed to PrEP (*P* < 0.01). M184V mutation was harboured more commonly in the recent PrEP use group (30% vs. 1%, *P* < 0.01). The proportion of individuals recently exposed to PrEP among those diagnosed with HIV rose sharply, reaching 21% in the first semester of 2020. Viral suppression was achieved by all patients intensified from PrEP to antiretroviral treatment (ART) who remained in care at week 24.

**Discussion:**

Rapid PrEP intensification to ART allowed to achieve high rates of HIV viral suppression despite significant rates of M184V mutation harboured in those newly diagnosed with HIV and reporting recent PrEP exposure.

## Introduction

HIV combination prevention strategies consisting of condom use, access to HIV preexposure prophylaxis (PrEP), postexposure prophylaxis and treatment-as-prevention with antiretroviral treatment (ART), contributed to the fall in new HIV diagnoses in men having sex with men (MSM) reported globally [1–3].

Oral PrEP as tenofovir disoproxil-fumarate (TDF)/emtricitabine (FTC) has been approved for a daily or ‘event-based’ dosing (EBD) around the time of a potential exposure to HIV. Its effectiveness in preventing HIV has been attested at 86% in trials [4,5] and as high as 95% when adherence is optimal [6].

HIV seroconversion in PrEP users is infrequent and its prevalence has been estimated at around 3% [7], with most PrEP ‘failures’ attributed to poor adherence. Less frequently, HIV breakthrough infections have been observed in those with good adherence to PrEP where the individual was already HIV positive but undiagnosed at PrEP initiation or even more rarely, due to the transmission of a HIV strain carrying resistance to PrEP components [7]. HIV seroconversion in individuals taking PrEP may be delayed [8], making the diagnosis of HIV challenging and on-going PrEP exposure in these individuals could lead to the development of HIV drug-resistance mutations [7,9]. Given the paucity of data on the management of patients in this setting, we analysed the proportion of individuals reporting recent exposure to PrEP at HIV diagnosis over time and reported data on possible reasons for PrEP failure, baseline genotypic resistance and their clinical management.

## Methods

Case-notes analysis of individuals diagnosed with HIV at 56 Dean Street (56DS), a combined sexual health and HIV service based in Soho, London, UK, over the 5-year period between 1 January 2016 and 31 December 2020 was performed from electronic patient records. In the absence of oral PrEP provision in the National Health Service (NHS) in UK, in January 2016 our clinic began offering free monitoring for those self-sourcing oral PrEP, in agreement with national PrEP guidance [10]. In October 2017, NHS England commissioned PrEP free of charge to sexual health clinic attendees through enrolment in the IMPACT trial. Recent PrEP exposure was defined as the self-reported PrEP intake in the 90 days prior the HIV diagnosis. PrEP adherence was defined as inadequate when three or fewer tablets per week were taken whilst on a daily regimen or when there was no compliance to the EBD scheme. Demographic data, immuno-virological parameters, and genotypic viral resistance test assay (GRT) results at diagnosis and PrEP sourcing were collected as well as the most recent HIV negative result, data on recent HIV infection using a testing algorithm (RITA) [11], timing and choice of antiretroviral treatment started and treatment outcomes. Individuals reporting recent/on-going PrEP exposure and who tested positive for HIV via rapid point-of-care-testing (POCT) and rapid dual testing of HIV p24-antigen/HIV antibodies were routinely offered to intensify PrEP to full ART by adding a third antiretroviral agent on the HIV diagnosis day. The choice of a third antiretroviral was at physician's discretion, however most individuals were offered either boosted-darunavir or a high genetic-barrier integrase-inhibitor (bictegravir or dolutegravir) when the former was contraindicated (i.e., due to potential drug-drug interactions). When HIV was detected via enzyme immunoassay (EIA) blood testing, individuals were advised not to discontinue PrEP and attend the clinic for intensification to full ART while confirmation of the HIV status via serum HIV-RNA was performed.

Recent HIV infection was defined as an initial positive HIV-EIA blood test result, preceded by a documented negative result within 6 months and/or having a positive RITA result.

Patients were defined as retained into HIV care if attendance to our service was recorded at 12 weeks or more after ART initiation. HIV viral suppression has been set at a HIV viral load target of <200 copies/ml within week 24 from diagnosis. We reported resistance-associated mutations only when carrying a degree of resistance to one or more antiretrovirals of current use [12]. Resistance to integrase-inhibitors was not routinely tested, therefore results are not included. Statistical analysis was performed on STATA 13.0 with Mann–Whitney *U*-test and Fisher's exact test calculations, used to measure differences between groups for continuous variables and for categorical variables, respectively.

## Results

**Fig. 1:**
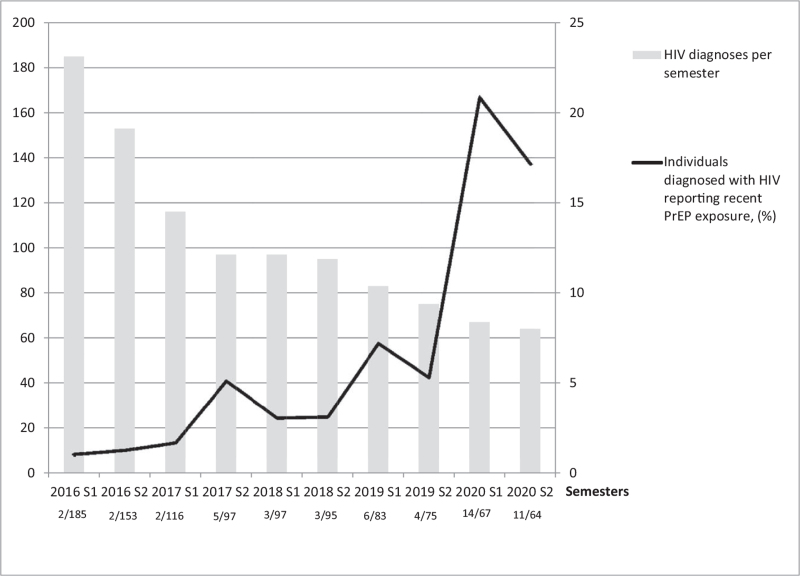
Trends of HIV diagnoses in MSM at 56 Dean Street (grey bars, *n*), each semester from 2016 to 2020 and rates of MSM newly diagnosed with HIV reporting recent PrEP exposure (black line, values expressed as a %).

A total of 1030 individuals (986 MSM, 96%) were diagnosed with HIV at 56DS over the period considered and 52/1030 (5%) of the HIV positive individuals reported recent PrEP use. Full demographics and baseline characteristics are displayed in Table 1. We found that 36/52 (69%) were recently infected with HIV (likely an underestimate, as 8/52 (15%) did not have a recent HIV test or a valid RITA result). The median time from the last negative HIV test was 159 days (interquartile range [IQR] 100–285 days). Whilst new HIV diagnoses declined over the five years (Fig. 1), the proportion of those newly diagnosed with HIV reporting recent PrEP use increased, reaching 21% in the first semester of 2020 and 17% in the second. The median time between the reported last PrEP pill and the HIV positive test was 14 days (IQR 1–48 days), with 18/52 (35%) reporting having taken PrEP the day before they tested HIV positive. PrEP consumption varied with 10/52 (19%) having switched between daily and event-based dosing at least once since their last HIV negative test. 36/52 (69%) were predominantly following the daily PrEP intake scheme at time of HIV diagnosis, while 16/52 (31%) were taking event-based dosing. 33/52 (63%) were sourcing PrEP outside a clinical setting (either online or through ex-partners or friends supply), 15/52 (29%) received PrEP within a clinical setting and in 4/52 (8%) the source was not reported.

**Table 1:** Baseline characteristics, immuno-virological characteristics and genotypic resistances in individuals newly diagnosed with HIV according to recent exposure to PrEP status.

Baseline characteristics	HIV+ reporting recent PrEP exposure, *n* = 52	HIV+ with no recent PrEP exposure, *n* = 978	*P*
Age, years (IQR)	34 (28–42)	32 (27–40)	0.52
MSM, *n* (%)	51 (98)^a^	935 (95)	0.42
White ethnicity*, n* (%)	34 (65)	644 (66)	0.54
UK-born, *n* (%)	18 (35)	326 (33)	0.88
Baseline HIV viral load, log_10_*n* (IQR)	3.48 (2.56–4.88)	4.72 (4.16–5.36)	**<0.01**
Baseline CD4^+^ cell count, mmc (IQR)	632 (468–886)	474 (347–655)	**<0.01**
Individuals with a HIV viral load <200 copies/ml at baseline, *n* (%)	10 (19)	32 (3)	**<0.01**
Genotypic resistance test at baseline, *n* (%)			**<0.01**
- Not performed	0	41 (4)	
- Unable to amplify viral DNA	9/52 (17)	27/939 (3)	
- Performed, with DNA amplification	43/52 (83)	912/939 (97)	
PrEP-related NRTI mutations, *n* (%)^b^			**<0.01**
- M184V/I	13 (30)	5 (1)	
- K65R	0	1 (<1)	
Other NRTI mutations, *n* (%)^b^			0.11
- L74V	1 (2)	0	
- M41L and/or L210W and/or T215Y/F	1 (2)	4 (<1)	
- D67N and/or K70R and/or K219Q/E/R/N	0	7 (1)	
NNRTI mutations, *n* (%)^b^			0.54
- K103N/S	3 (7)	29 (3)	
- V108V/I	1 (2)	8 (1)	
- E138A	0	26 (3)	
- G190A/S/E	1 (2)	0	
PI mutations, *n* (%)^b^			0.62
- M46I/L	0	6 (1)	
- L90M	0	18 (2)	

IQR, interquartile range; MSM, men having sex with men; NNRTI, non-nucleoside reverse transcriptase inhibitors; NRTI, nucleoside reverse transcriptase inhibitor; PI, protease inhibitor; PrEP, preexposure prophylaxis.

a One individual identified themselves as a trans-woman.

b % are calculated taking into account only individuals where viral DNA was amplified.

Recent PrEP exposure was defined as a self-reported PrEP intake in the 90 days prior the HIV diagnosis. Fractions report numbers of people newly diagnosed with HIV reporting recent PrEP exposure/total number of HIV diagnoses per semester. MSM, men having sex with men; PrEP, preexposure prophylaxis.

When reasons leading to PrEP failure were investigated, 24/52 (46%) reported inadequate adherence (11 on a daily regimen, 13 on EBD), 9/52 (17%) had issues with PrEP supply resulting in its discontinuation, 5/52 (10%) interrupted PrEP after starting a monogamous relationship, 3/52 (6%) did not plan to have sex, 3/52 (6%) chose to interrupt PrEP and 1/52 (2%) was taking an antiretroviral regimen not licensed for PrEP (raltegravir). A reason for PrEP failure could not be identified in 7/52 (13%) who reported excellent adherence to PrEP from their last HIV-negative test to the time of their HIV diagnosis. Baseline HIV viral load was significantly lower in those with recent PrEP exposure (*n* = 52) than in those not reporting recent PrEP intake (*n* *=* 978), whilst baseline CD4+ cell count was higher in the PrEP-exposed group. GRT was less likely to be carried out successfully in individuals reporting recent PrEP exposure than those not exposed to PrEP (83% vs. 97%, *P* < 0.01) (full display of mutations shown in Table 1). Among those harbouring a M184 V/I mutation, no differences between reported PrEP intake (daily vs. EBD) were found, although 10/13 (77%) reported sub-optimal adherence to either regimen. The median time from the reported last PrEP pill intake to the HIV diagnosis was 11 days (IQR 1–54 days).

Management of individuals diagnosed with HIV with recent PrEP use was examined: 48/52 (92%) individuals attended a medical appointment to start full ART, with 44/48 (92%) deciding to start treatment immediately. Four individuals transferred their care elsewhere before attending this appointment. 47/52 (90%) patients were successfully retained in subsequent HIV care at 24 weeks and all started ART by then. Median time from positive HIV testing to ART initiation was 8 days (IQR 6–14 days). All patients were started on a tenofovir-based backbone ART, whilst the third agent of choice was boosted-darunavir (*n* *=* 28), bictegravir (*n* = 8), dolutegravir (*n* = 7) or raltegravir (*n* = 4). 12/28 (43%) were then switched off boosted-darunavir in favour of integrase-inhibitor combinations within 3 months from ART initiation as per clinic protocol [13]. 39/42 (93%) individuals had a baseline HIV viral load >200 copies/ml at HIV diagnosis and were followed up at 24 weeks (two transferred their care elsewhere and one was lost at follow-up after an initial assessment). All 39 individuals achieved viral suppression at 24 weeks (*n* = 35 treated with either dolutegravir, bictegravir or boosted-darunavir and *n* = 4 with raltegravir). 9/52 (17%) individuals had unsuccessful GRT DNA amplification; six were intensified with boosted-darunavir, two with dolutegravir and one transferred his care abroad. All eight individuals on ART maintained a suppressed HIV viral load at week 24.

## Discussion

The implementation of a comprehensive strategy combining easy access to HIV testing, immediate ART offer and expanded PrEP access likely explains the decline in HIV infections among MSM witnessed in the past 5 years [14]. HIV infection is uncommon when PrEP is used correctly, however we observed a sharp rise in the proportion of MSM diagnosed with HIV who reported recent exposure to PrEP in 2020. This was mostly associated with sup-optimal adherence to the chosen PrEP scheme and we suggest this trend may be continuing with wider availability of PrEP and increasing numbers of PrEP users. We confirm high rates of acquired viral resistance to emtricitabine, mostly in the form of M184V/I mutation, harboured in almost a third of these cases. Resistance mutations to PrEP components is predominately acquired through unrecognized or delayed HIV seroconversion before PrEP start, with the development of resistance observed in 45.8% of cases and higher prevalence among those who are adherent to PrEP [7]. A less common pathway for PrEP failure and resistance development is through HIV breakthrough due to sub-optimal PrEP adherence, which is believed to lead to resistance in about 5% of cases [7].

At least two-thirds of those carrying a M184V/I mutation in our cohort acquired HIV recently, with a large majority reporting sub-optimal PrEP adherence, irrespectively of the PrEP scheme adopted, confirming the trend observed previously in our cohort [15] and in other reports [16].

The number of individuals acquiring HIV and reporting recent exposure to PrEP is expected to grow further with the increase of PrEP users. This often comes with delayed HIV seroconversions, challenging the current assays and posing a diagnostic challenge. Lower rates of viral DNA amplification and less information on transmitted resistance mutations harboured by the virus represent a further challenge for the clinician, having to select an optimal ART to start. Minimizing the possibility of undiagnosed HIV infection through an accurate HIV testing strategy at PrEP start is required to curtail the risk of antiretroviral resistance development [17].

We routinely offered full ART intensification on the day of HIV diagnosis: ART should contain at least two active agents against HIV, of which one with a high genetic barrier such as boosted-darunavir or the newer integrase inhibitors (dolutegravir or bictegravir) [18]. Intensification did not affect subsequent adherence to ART or engagement into HIV care, with high rates of virological suppression at week 24 from ART start. Like previous reports, median HIV viral loads at baseline were lower in those with recent PrEP exposure [9,19] and a suppressed viral load at baseline was more common in this group than those who were PrEP naïve.

We acknowledge that our data on reported PrEP use may be subject to recall and social desirability biases from patients. Poor adherence to PrEP might also have been under reported. As a retrospective study, we are also aware that the data collected might be affected by an information bias; however we believe this model of care could be translatable in other healthcare contexts with similar patient cohorts. PrEP fatigue, leading to sub-optimal PrEP adherence and subsequently discontinuation is an increasingly documented phenomenon [20]. This could impact in the future frequency of clinically relevant resistance, as numbers of individuals exposed to PrEP increase. Patient education and widening the array of PrEP options on offer (for example the use of long-acting formulations such a cabotegravir) should help address this. With the rapid expansion of PrEP use in MSM, we expect an overall fall in the number of HIV diagnoses. Nonetheless, Prospective cohort studies are needed to monitor the frequency of clinically relevant resistance and the long term outcomes, in order to inform optimal ART intensification which remains the strategy of choice for new HIV diagnoses in those who have and have not taken PrEP.

## Acknowledgements

### Conflicts of interest

There are no conflicts of interest.

Funding: SMcC was supported by the Medical Research Council (MC_UU_00004/03).

56 Dean Street Collaborative Group is comprehensive of: Keerti Gedela, Diarmuid Nugent, Sheel Patel and Tara Suchak.
